# Machine learning regression algorithms to predict short-term efficacy after anti-VEGF treatment in diabetic macular edema based on real-world data

**DOI:** 10.1038/s41598-023-46021-2

**Published:** 2023-10-31

**Authors:** Ruijie Shi, Xiangjie Leng, Yanxia Wu, Shiyin Zhu, Xingcan Cai, Xuejing Lu

**Affiliations:** 1 Department of Ophthalmology, Eye College of Chengdu University of Traditional Chinese Medicine, Chengdu, 610000 Sichuan China; 2 Ineye Hospital of Chengdu University of Traditional Chinese Medicine, Chengdu, 610000 Sichuan China; 3 Key Laboratory of Sichuan Province Ophthalmopathy Prevention & Cure and Visual Function Protection with Traditional Chinese Medicine, Chengdu, 610000 Sichuan China; 4 Retinal Image Technology and Chronic Vascular Disease Prevention & Contro and Collaborative Innovation Center, Chengdu, 610000 Sichuan China

**Keywords:** Eye diseases, Computer science

## Abstract

The objective of this retrospective study was to predict short-term efficacy of anti-vascular endothelial growth factor (VEGF) treatment in diabetic macular edema (DME) using machine learning regression models. Real-world data from 279 DME patients who received anti-VEGF treatment at Ineye Hospital of Chengdu University of TCM between April 2017 and November 2022 were analyzed. Eight machine learning regression models were established to predict four clinical efficacy indicators. The accuracy of the models was evaluated using mean absolute error (*MAE*), mean square error (*MSE*) and coefficient of determination score (*R*^2^). Multilayer perceptron had the highest *R*^2^ and lowest *MAE* among all models. Regression tree and lasso regression had similar *R*^2^, with lasso having lower *MAE* and *MSE*. Ridge regression, linear regression, support vector machines and polynomial regression had lower *R*^*2*^ and higher *MAE*. Support vector machine had the lowest *MSE*, while polynomial regression had the highest *MSE*. Stochastic gradient descent had the lowest *R*^2^ and high *MAE* and *MSE*. The results indicate that machine learning regression algorithms are valuable and effective in predicting short-term efficacy in DME patients through anti-VEGF treatment, and the lasso regression is the most effective ML algorithm for developing predictive regression models.

## Introduction

Diabetes mellitus (DM) is a systemic disease associated with various health problems, which lower life quality and cause a higher mortality rate among patients^1^. According to a global report on diabetes by World Health Organization (WHO), there were 451 million adults with DM worldwide in 2017 and this number was expected to increase to 693 million by 2045^2^. Moreover, almost half of the population (49.7%) may live with undiagnosed DM. Diabetic retinopathy (DR) is a specific microvascular complication of DM, which has emerged as the primary cause of vision loss in the general population across various countries^3–6^.

Diabetic macular edema (DME) is typified by retinal thickening, hard exudates, microaneurysms, and macular hemorrhage, can manifest at any stage of DR and often results in severe vision loss^7–9^. In developing countries, the risk of vision loss is higher than developed country due to disparities in income levels and medical conditions^10^. The etiology and pathogenesis of DME are multifaceted. A critical factor in the pathogenesis of DR is the hyperglycemic state of DM, which triggers microangiopathy by upregulating various inflammatory and angiogenic mediators, particularly vascular endothelial growth factor (VEGF), in the vascular endothelium. These mediators induce pathological alterations in the blood-retinal barrier, resulting in fluid leakage into the extracellular space^11^, leading to macular edema. Consequently, anti-VEGF therapy is a powerful and effective treatment method against DME^12^ .

Vitreous cavity injection of anti-VEGF drugs is believed as standard treatment options which can greatly save the patient's visual acuity (VA)^13–16^. However, anti-VEGF therapy can cause adverse events such as cataract formation, elevated intraocular pressure, retinal artery occlusion, ocular hemorrhage, sterile intraocular inflammation, infectious endophthalmitis^17,18^. Meanwhile, anti-VEGF therapy is expensive and requires repeated treatments. Costs for treatment over a 5-year period were reported in the United States for diabetic retinopathy treated with anti-VEGF and estimated to be $40,825, which may pose a huge financial burden on patients^19^. Meanwhile, not all patients respond equally to the standard treatment of intravitreal therapy^20^. Therefore, predicting short-term efficacy after anti-VEGF treatment could have great clinical and economic significance.

Artificial intelligence (AI) is one of the most popular topics today and has been widely used in autonomous driving, face recognition, and intelligent robotics^21,22^. Machine learning (ML) is a branch of AI which aims to learn patterns from data to improve performance in various tasks^23^. Compared to traditional statistical methods, ML is more efficient, less susceptible to human factors, and can handle complex data. ML methods have been used extensively to solve various complex challenges in recent years in medical areas, such as prediction of disease efficacy, medical image processing, disease risk prediction, etc.^24–26^. However, most of the above applications adopted classification algorithms, while the application of the regression algorithm is currently not developed well. Compared to the classification algorithm, the regression algorithm can handle continuous variables with higher precision and broader applicability, which confers it a strong potential and value for clinical development.

At present, the application of ML in DR, particularly in DME, is predominantly concentrated in the realm of diagnostics. The exploration of therapeutic effect predictions, which are based on regression algorithms, is still in the nascent stage. For DME, the implementation of an ML regression prediction model has the potential to furnish doctors and patients with more precise predictive data. This, in turn, can aid in clinical decision-making processes, mitigate clinical risks, and curtail superfluous treatment expenses. Consequently, the utilization of ML for prognostic predictions of DME has great clinical and economic value. In light of these considerations, the objective of this study is to establish ML regression models using real-world data, to predict the short-term efficacy of anti-VEGF treatment for DME.

## Methods

All methods were carried out in accordance with all relevant guidelines and regulations, and the study was reviewed and approved by the Academic Committee and the Ethics Committee of Ineye Hospital of Chengdu University of Traditional Chinese Medicine (Ethics number: 2022yh-023). This study has obtained informed consent from all subjects or their legal guardian(s).

### Source of data and participants

Patients diagnosed with DME in Ineye Hospital of Chengdu University of Traditional Chinese Medicine from April 2017 to November 2022 were included. After preparing valid data, ML was used to predict the short-term efficacy of DME patients after anti-VEGF treatment.

The inclusion criteria for the patients were: (1) clinical diagnosis of DME based on the Diabetic Retinopathy Preferred Practice Pattern 2019^27^; (2) receipt of at least one anti-VEGF treatment; (3) age between 18 and 85 years; (4) follow-up period of no more than 3 months. The exclusion criteria were: (1) the presence of other eye disorders that may affect VA, such as glaucoma, age-related macular degeneration (AMD), retinal detachment, etc.; (2) lack of clinical data; (3) refractive interstitial opacification obscuring the macula; (4) undergoing intraocular surgery.

### Data collection

We collected data including sex, age, type of anti-VEGF drugs, follow-up time, best corrected visual acuity (BCVA), intraocular pressure (IOP), central subfield thickness (CST), cube volume (CV), cube average thickness (CAT), macular thickness divided by ETDRS grid^28^ (center, inner superior (IS), inner temporal (IT), inner inferior (II), inner nasal (IN), outer superior (OS), outer temporal (OT), outer inferior (OI), outer nasal (ON), and ganglion cell thickness (GC) analysis (average, minimum, superior, temporal superior, nasal superior, nasal inferior, inferior, temporal inferior). We included both the first and the second visits of BCVA, CST, CV, and CAT as the clinical predictors.

### Data preprocessing

BCVA was converted from decimal notation to LogMAR notation for statistical analysis. Count finger, hand movement and light perception were recorded as 2.0, 2.3 and 2.6 in LogMAR notation respectively^29^. No light perception should be recorded as infinity in LogMAR notation, for calculation, was recorded as 100 in LogMAR notation. 514 missing data were complementary by IBM SPSS statistics 27 using methods of regression to adjust residuals.

### Machine learning models and training

We established eight ML regression models, including linear regression, polynomial regression, ridge regression, lasso regression, support vector machines (SVM), regression tree, multilayer perceptron (MLP) and stochastic gradient descent (SGD) regression. Using Python 3.9.0 and Scikit-learn 1.2.0 for training and testing. We used Matplotlib 3.5.2 to draw the figures. We split the data into 75% for training and 25% for testing.

Linear models are regression methods that assume a linear relationship between the features and the target. Ordinary Least Squares is a linear model that minimizes the sum of squared errors between the observed and predicted targets^30^. Polynomial regression models y as an nth-degree polynomial in x. It can capture nonlinear relationships. It was influential in regression analysis history, with a focus on design and inference^31^. Ridge regression is a method of estimating the coefficients of multiple-regression models in scenarios where the independent variables are highly correlated^32^. It regularizes ill-posed problems and reduces multicollinearity in linear regression^33^. In general, the method provides improved efficiency in parameter estimation problems in exchange for a tolerable amount of bias^34^. Lasso is a linear model with sparse coefficients. It reduces the number of features and can recover the exact non-zero coefficients under certain conditions^30^. SVM is a young and practical branch of statistical learning theory. It transforms low-dimensional nonlinear functions into high-dimensional spaces via a smart nonlinear mapping, without requiring its explicit form^35,36^. Classification and regression trees are nonparametric regression methods that recursively partition the feature space into rectangular areas^37^, first proposed by Breiman et al.^38^. They use binary recursive partitioning to split the data into smaller groups along each branch^37^. MLP is a feedforward artificial neural network (ANN) with full connectivity and at least three layers: input, hidden and output. Each node except the input ones is a nonlinear neuron. MLP uses backpropagation for supervised learning^39,40^. It differs from a linear perceptron by its multiple layers and non-linear activation. It can handle non-linearly separable data^41^. SGD is a simple yet very efficient approach to fit linear models. It is particularly useful when the number of samples (and the number of features) is very large. The method allows online/out-of-core learning^30,42^.

Python 3.9.0 and Scikit-learn 1.2.0 were used for training and testing. Matplotlib 3.5.2 was used to draw the figures. The dataset was split into training dataset (75%) and testing dataset (25%).

### Evaluating the performance of prediction models

Mean absolute error (*MAE*), mean square error (*MSE*), and coefficient of determination (*R*^2^) score were used as the evaluation metrics to assess the accuracy of the prediction models.

*MAE* is a risk metric that corresponds to the expected value of the absolute error loss or -norm loss $l1$. On the other hand, *MSE* is a risk metric that corresponds to the expected value of the squared (quadratic) error or loss.* R*^2^ value represents the proportion of variance (of $y$) that can be explained by the independent variables in the model, which indicates goodness of fit. A lower value of *MAE* and *MSE* indicates a higher accuracy of the prediction model. Conversely, a higher *R*^2^ score, with the best possible score being 1.0, suggests a better fit of the prediction model. It’s worth noting that the *R*^2^ score can be negative if the model’s performance is arbitrarily worse. Therefore, a score closer to 1 indicates a better fit of the prediction model^30,42^.

For a sample of n observations $y$ (${y}_{i}$ , $i$= 1,2, . . .,n) and n corresponding model predictions $\widehat{y}$, the *MAE*, *MSE* and* R*^*2*^ score are

$$MAE=\frac{1}{n}\sum_{i=1}^{n}\left|{y}_{i}-{\widehat{y}}_{i}\right|$$

$$MSE=\frac{1}{n}\sum_{i=1}^{n}{\left({y}_{i}-{\widehat{y}}_{i}\right)}^{2}$$

$${R}^{2}=1-\frac{\sum_{i}{\left({\widehat{y}}_{i}-{y}_{i}\right)}^{2}}{\sum_{i}{\left({\overline{y} }_{i}-{y}_{i}\right)}^{2}}$$

Weighted sum of *MSE*, *MAE* and* R*^*2*^ score in BCVA, CAT, CST and CV for each model to tally the final score.

### Data correlation

Regression coefficients (coef) are estimates of the unknown population parameters and describe the relationship between a predictor variable and the response. Which can be shown in four models (linear regression, ridge regression, lasso regression and SGD). The larger the absolute value of coef, the higher the correlation between the predictor variable and the response variable. A positive coef value indicates a positive correlation with the predicted outcome, while the opposite is a negative correlation.

### Ethics approval

This study was reviewed and approved by the Academic Committee and the Ethics Committee of Ineye Hospital of Chengdu University of Traditional Chinese Medicine (Ethics number: 2022yh-023).

## Results

### Characteristics of dataset

279 eyes were included in our research, the overall characteristics of the dataset are shown in Table 1.

**Table 1:** Baseline of characteristics.

Characteristics	Value	
Age, mean ± SD, years	58.53 ± 11.546	
Sex, no. (%)		
Male	173 (62)	
Female	106 (38)	
Type of anti-VEGF drugs, no. (%)		
Ranibizumab	60 (22.5)	
Conbercept	187 (67)	
Aflibercept	32 (11.5)	
Follow-up period, mean ± SD, days	32.16 ± 23.011	
OCT, no. (%)		
CarlZeissMeditec	117(41.9)	
SVisionImaging	162(58.1)	
BCVA, mean ± SD	2.55 ± 13.196	
CST, mean ± SD, μm	372.609 ± 158.616	
CV, mean ± SD, mm^3^	14.425 ± 5.529	
CAT, mean ± SD, μm	369.597 ± 74.529	
IOP, mean ± SD, mm Hg	16.29 ± 3.777	
Macular thickness (ETDRS grid), mean ± SD, μm		
Center	386.92 ± 152.966	
IS	419.078 ± 113.287	
IT	418.482 ± 127.267	
II	409.239 ± 108.215	
IN	415.559 ± 108.534	
OS	380.325 ± 100.977	
OT	375.792 ± 109.632	
OI	353.743 ± 90.237	
ON	388.140 ± 93.757	
Ganglion cell layer thickness, mean ± SD, μm		
Average	80.615 ± 26.761	
Temporal superior	66.861 ± 35.369	
Superior	82.023 ± 31.79	
Nasal superior	86.159 ± 37.422	
Nasal inferior	83.378 ± 37.38	
Inferior	74.009 ± 29.414	
Temporal inferior	75.886 ± 33.86	
Minimum	42.639 ± 36.302	

### Model performance on various clinical indicators

**Figure 1:**
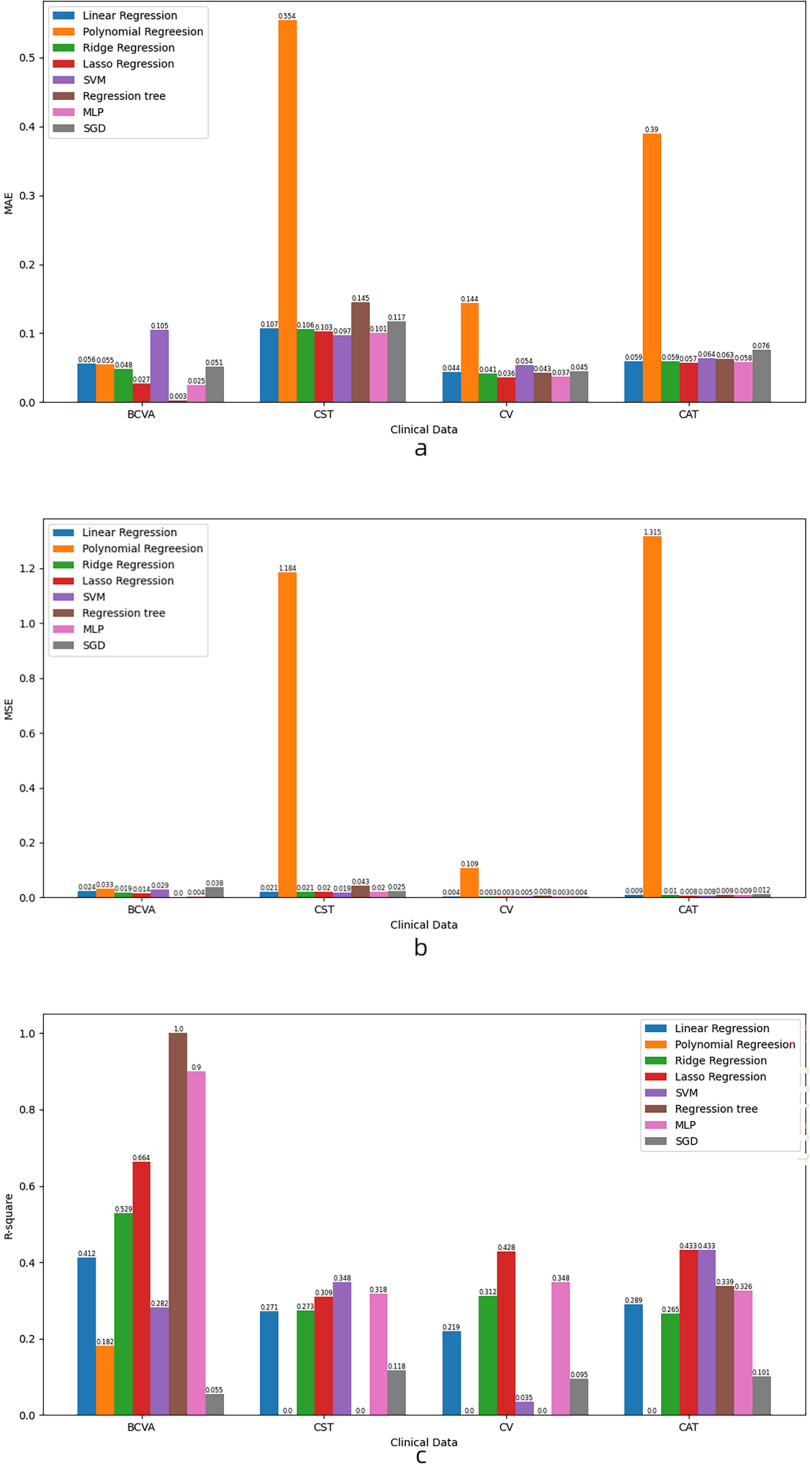
Model performance on the test set. (**a**) Performance of models with *MAE* as evaluation index. (**b**) Performance of models with *MSE* as evaluation index. (**c**) Performance of models with *R*^2^ as evaluation index.

The results are shown in Table 2. For BCVA, the best model is regression tree, which has zero *MAE* and *MSE* and one *R*^*2*^ for both sets. The worst model is SVM, which has the highest *MAE* and *MSE* for both sets. The other models have similar performance on the training set but vary on the testing set. For CST, the best model is lasso regression, which has the lowest *MAE* and *MSE* and the highest *R*^2^ for both sets. The worst model is polynomial regression, which has very high *MAE* and *MSE* and negative *R*^2^ for the testing set. The other models have similar performance on both sets. For CV, the best model is MLP, which has the lowest *MAE* and *MSE* and the highest *R*^2^ for both sets. The worst model is polynomial regression, which has very high *MAE* and *MSE* and negative *R*^2^ for the testing set. The other models have similar performance on both sets. For CAT, the best model is MLP, which has the lowest *MAE* and *MSE* and one of the highest *R*^2^ for both sets. The worst model is polynomial regression, which has very high *MAE* and *MSE* and negative *R*^2^ for the testing set. The other models have similar performance on both sets. Model performance on the test set is shown in Fig. 1.

**Table 2:** Model performance on various clinical indicators.

Clinical indicator	Model	Evaluation index					
		Training set			Testing set		
		MAE	MSE	R^2^	MAE	MSE	R^2^
BCVA	Linear regression	0.025	0.002	0.887	0.056	0.024	0.412
	Polynomial regression	0.000	0.000	1.000	0.055	0.033	0.182
	Ridge regression	0.022	0.002	0.874	0.048	0.019	0.529
	Lasso regression	0.015	0.004	0.768	0.027	0.014	0.664
	SVM	0.091	0.016	0.137	0.105	0.029	0.282
	Regression tree	0.000	0.000	1.000	0.003	0.000	1.000
	MLP	0.004	0.000	0.998	0.025	0.004	0.900
	SGD	0.029	0.017	0.086	0.051	0.038	0.055
CST	Linear regression	0.094	0.015	0.501	0.107	0.021	0.271
	Polynomial regression	0.000	0.000	1.000	0.554	1.184	− 40.008
	Ridge regression	0.094	0.015	0.500	0.106	0.021	0.273
	Lasso regression	0.098	0.016	0.458	0.103	0.020	0.309
	SVM	0.099	0.017	0.441	0.097	0.019	0.348
	Regression tree	0.000	0.000	1.000	0.145	0.043	− 0.478
	MLP	0.090	0.015	0.515	0.101	0.020	0.318
	SGD	0.122	0.024	0.198	0.117	0.025	0.118
CV	Linear regression	0.037	0.004	0.468	0.044	0.004	0.219
	Polynomial regression	0.000	0.000	1.000	0.144	0.109	− 22.028
	Ridge regression	0.037	0.004	0.462	0.041	0.003	0.312
	Lasso regression	0.035	0.005	0.373	0.036	0.003	0.428
	SVM	0.053	0.006	0.185	0.054	0.005	0.035
	Regression tree	0.000	0.000	1.000	0.043	0.008	− 0.678
	MLP	0.030	0.004	0.526	0.037	0.003	0.348
	SGD	0.036	0.006	0.191	0.045	0.004	0.095
CAT	Linear regression	0.042	0.004	0.660	0.059	0.009	0.289
	Polynomial regression	0.000	0.000	1.000	0.390	1.315	− 98.086
	Ridge regression	0.042	0.004	0.659	0.059	0.010	0.265
	Lasso regression	0.043	0.005	0.627	0.057	0.008	0.433
	SVM	0.053	0.006	0.558	0.064	0.008	0.433
	Regression tree	0.000	0.000	1.000	0.063	0.009	0.339
	MLP	0.032	0.003	0.786	0.058	0.009	0.326
	SGD	0.052	0.006	0.515	0.076	0.012	0.101

### Model performance

**Figure 2:**
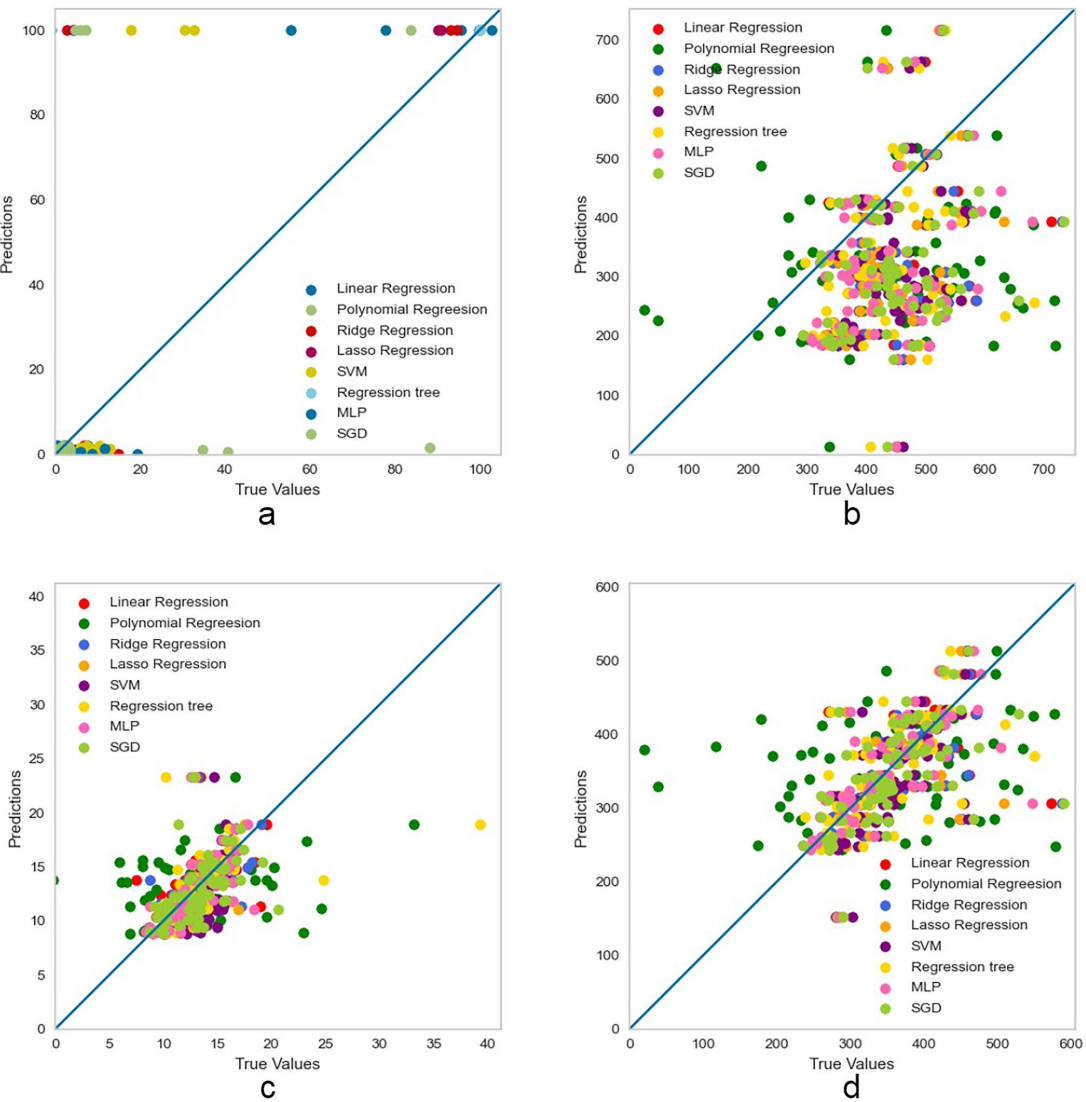
Visualization of prediction results. (**a**) Performance of each model when predicting BCVA, (**b**) performance of each model when predicting CST, (**c**) performance of each model when predicting CV, (**d**) performance of each model when predicting CAT.

The visualization of prediction results is shown in Fig. 2. MLP has the highest *R*^2^ (0.1626) and lowest *MAE* (0.0108). Regression tree and lasso regression have similar *R*^2^ (0.1433 and 0.1428), with lasso having lower *MAE* (0.0109) and *MSE* (0.0044). Ridge regression, linear regression, SVM and polynomial regression have lower *R*^2^ (0.1090, 0.0906, 0.0757 and 0.0228) and higher *MAE* (0.0116, 0.0118, 0.0122 and 0.0607). SVM has the lowest *MSE* (0.0043), while polynomial regression has the highest *MSE* (0.3930). SGD has the lowest *R*^2^ (0.0226) and high *MAE* (0.0133) and *MSE* (0.0059).

### Data correlation in prediction models

According to our results, the thickness of different areas of the macula and the type of anti-VEGF drug had a large impact on the predicted results. The specific results are as follows:

For BCVA prediction, the highest correlations for linear regression were CAT (0.765), Center (0.619), IS (−0.391) and OT (−0.386); for ridge regression were CAT (0.457), Center (0.286), IS (−0.243) and conbercept (0.232) ; for lasso regression were OT (−0.047), OS (−0.0420), sex (−0.009), and conbercept (0.005); for SGD were IOP (0.013), sex (−0.012), conbercept (0.011) and CV (0.009).

For CST prediction, the highest correlations for linear regression were Center (0.275), CV (−0.250), OI (−0.220) and IS (0.203); for ridge regression were conbercept (0.304), Center (0.281), ranibizumab (0.240) and CV (−0.235); for lasso regression were Center (0.189), CV (−0.188), ON (−0.096) and temporal superior (GC) (0.070); for SGD were conbercept (0.058), IS (0.056), Center (0.055) and IN (0.042).

For CAT prediction, the highest correlations for linear regression were OS (0.288), OT (0.213), temporal superior (GC) (0.165) and ON (0.158); for ridge regression were conbercept (0.260), OS (0.256), aflibercept (0.238) and ranibizumab (0.222); for lasso regression were OS (0.178), OT (0.127), temporal superior (GC) (0.072) and BCVA (0.006); for SGD were conbercept (0.083), OS (0.069), OI (0.070) and OT (0.067).

For CV prediction, the highest correlations for linear regression were CAT (0.295), IS (−0.287), Center (0.259) and conbercept (0.024); for ridge regression were conbercept (0.248), aflibercept (0.214), ranibizumab (0.207) and CAT (0.295); for lasso regression were CAT (0.082), temporal inferior (GC) (0.070), conbercept (0.032) and ON (−0.007); for SGD were conbercept (0.057), OI (0.039), OT (0.032) and OS (0.032). Complete results are shown in Supplementary 1.

## Discussion

In this study, we developed eight ML regression models to predict the therapeutic effect of DME patients after anti-VEGF treatment. Our results demonstrate that ML regression algorithms have a high potential for efficacy prediction in diseases. We observed that the overall performance of the eight ML models was better on BCVA than on the other three clinical indicators. A possible reason for this is that BCVA measurements were performed independently by a standard procedure, while other clinical indicators were obtained by two OCT machines that varied in algorithm, clarity, and scan interval. Among all models, MLP and lasso regression models outperformed others. MLP is a type of ANN, which is essential for building nonlinear relationship models in high-dimensional datasets^43^. MLP adds one or more hidden layers on top of the single-layer neural network, which can be iterated. Therefore, it has a greater capacity to learn and generalize, to fit multiple classes of functions, and to predict nonlinear data. Lasso regression has been widely applied in general regression models to predict the risk of likely outcomes^44^. Lasso regression performs both variable selection and regularization to improve the prediction accuracy and interpretability of the resulting model^45^. Thus, lasso regression might be more appropriate for our regression task.

Our results indicate that CAT and macular area thickness have a significant impact on BCVA prediction. DME can severely impair VA when retinal leakage accumulates in the OPL, which increases the overall macular thickness and may disturb the normal path of light from the inner retinal surface to the outer segments^46^. Moreover, DME is also associated with vascular changes that have been linked to the degree of visual impairment^47–50^. Meanwhile, CST prediction is more related to the overall macular thickness. In pathological conditions, altered organization and stability of junction proteins of RMG (e.g., ZO-1) can lead to cyst formation ^46,51^. RMG cell density is about five times higher in the fovea than in the periphery. Therefore, due to its thinness, the fovea is more susceptible to edema and has a greater influence on macular thickness^52^. The prediction of CAT and CV was more correlated with the thickness of the outer macular ring and ganglion cell layer because of their anatomical characteristics. The outer macula has a thick layer of ganglion cells while the central macular recess is the thinnest part of the entire macula^46^. This may result in a higher likelihood of volume change in the outer macula affecting the thickness of different areas of the macula.

We also found that the type of anti-VEGF drugs was highly correlated with the predicted outcomes of BCVA, CST, CAT, and CV. Conbercept had a greater impact on these outcomes than ranibizumab and aflibercept. Both aflibercept and conbercept are recombinant decoy receptor groups of VEGF^53^. They block free VEGF-mediated signalling through their cognate receptors, thus inhibiting the pro-inflammatory, hyperpermeable, and pro-angiogenic effects of VEGF in a similar way. However, aflibercept showed greater therapeutic effects than conbercept, including more VA improvement and anatomical recovery. It also had advantages over mAb drugs (e.g., bevacizumab and ranibizumab) in enhancing VA and reducing macular edema^54^. Nevertheless, aflibercept is the most expensive among anti-VEGF drugs and may have low patient acceptability due to the high cost. Conbercept is a novel multi-targeted anti-VEGF drug that has demonstrated excellent efficacy in treating patients with DME and neovascular AMD. The induction of domain 4 distinguishes conbercept from aflibercept and enhances its VEGF binding capacity. Due to the structural change, conbercept may affect steadily in the vitreous humour^55^. Conbercept requires fewer injections, has a lower risk of injection-related complications, and is more cost-effective than ranibizumab^54,56^. Among the included patients, most of them chose conbercept. This may have been driven by financial considerations and better outcomes. However, this also introduces some degree of bias. The better efficacy for DME and the bias may have contributed to the higher predictive relevance of conbercept compared to other anti-VEGF drugs.

Several studies^57–60^ used classification algorithms with less than six models. We used regression algorithms innovatively and established up to eight models. However, the evaluation indicators of classification and regression algorithms are different, so we cannot directly compare their accuracy. We hope that future research will explore more on the regression algorithm. Moreover, most of these studies used public datasets as their data sources, while we used a self-built database based on real-world data. Therefore, our model may be more consistent with the current state of the population and have epidemiological significance.

Some limitations should be drawn out. First, we had limited access to patient information, such as blood glucose, blood pressure, insulin use, smoking history, family history, etc.^61^ , that was not available in our outpatient system. This may have led to less input for model training and reduced model accuracy. We imputed missing data using regression methods, but this may differ from the actual data and affect the prediction results. Second, polynomial regression and regression trees showed overfitting due to a large number of parameters and complex structure. Both polynomial regression and regression trees are prone to overfitting because they both increase the complexity and degrees of freedom of the model which may learn too many features that do not reflect the general patterns of the data.

The application of artificial intelligence (AI) in the medical field is currently not mature enough. Machine learning (ML), as a subset of AI, is commonly used for classification tasks, while regression tasks, which are commonly used for quantitative analysis, are rarely addressed. However, ML regression algorithms have potential in clinical settings and the public health field, such as developing treatment plans and predicting post-operative complications^62,63^. For example, for diabetic macular edema (DME) patients, clinicians can select the most suitable anti-VEGF drug by considering ML-predicted indicators and drug prices, The improved model can be better applied to medical institutions in underdeveloped areas and primary hospitals, and enhance the diagnostic efficiency and accuracy of clinicians, as well as help reduce the economic burden on patients. With the rapid development of computer science, deep learning has emerged as an extension of ML that is commonly used for prediction and classification tasks using images in the medical field. Combining ML regression algorithms with deep learning can greatly expand the type and amount of data available to make more accurate clinical predictions, this will help improve the medical level in underdeveloped areas and greatly reduce the pressure on clinicians. In general, AI will inevitably change the existing mode of clinical practice in the future. Although AI may not replace physicians, the “AI + physician” mode of diagnosis and treatment may not be far away.

## Conclusion

ML regression algorithms are effective in predicting the short-term efficacy of anti-VEGF treatment in DME patients, which are valuable in clinical and public health settings. Our results show that BCVA has the best prediction result compared to CST, CAT and CV. Furthermore, our analysis suggests that the lasso regression algorithm is the most effective ML technique for developing predictive regression models.

### Supplementary Information

Supplementary Information 1.

Supplementary Information 2.

## Data Availability

The raw data is in Supplementary 2, and other data are available from the corresponding author on reasonable request.
